# Inheritance of the reduced mitochondria of *Giardia intestinalis* is coupled to the flagellar maturation cycle

**DOI:** 10.1186/s12915-021-01129-7

**Published:** 2021-09-07

**Authors:** Pavla Tůmová, Luboš Voleman, Andreas Klingl, Eva Nohýnková, Gerhard Wanner, Pavel Doležal

**Affiliations:** 1 Institute of Immunology and Microbiology, First Faculty of Medicine, Charles University, Prague, Czech Republic; 2 Department of Parasitology, Faculty of Science, Charles University, BIOCEV, Vestec, Czech Republic; 3 Plant Development and Electron Microscopy, Department of Biology I, Biocenter of Ludwig-Maximilians University, Munich, Germany; 4 Department of Biology I, Biocenter of Ludwig-Maximilians University, Munich, Germany

**Keywords:** Mitochondrial inheritance, Mitosomes, mitochondrial evolution, Flagellum, Cytoskeleton, Cell cycle, Mitochondrial division, Protist, *Giardia*

## Abstract

**Background:**

The presence of mitochondria is a distinguishing feature between prokaryotic and eukaryotic cells. It is currently accepted that the evolutionary origin of mitochondria coincided with the formation of eukaryotes and from that point control of mitochondrial inheritance was required. Yet, the way the mitochondrial presence has been maintained throughout the eukaryotic cell cycle remains a matter of study. Eukaryotes control mitochondrial inheritance mainly due to the presence of the genetic component; still only little is known about the segregation of mitochondria to daughter cells during cell division. Additionally, anaerobic eukaryotic microbes evolved a variety of genomeless mitochondria-related organelles (MROs), which could be theoretically assembled de novo, providing a distinct mechanistic basis for maintenance of stable mitochondrial numbers. Here, we approach this problem by studying the structure and inheritance of the protist *Giardia intestinalis* MROs known as mitosomes.

**Results:**

We combined 2D stimulated emission depletion (STED) microscopy and focused ion beam scanning electron microscopy (FIB/SEM) to show that mitosomes exhibit internal segmentation and conserved asymmetric structure. From a total of about forty mitosomes, a small, privileged population is harnessed to the flagellar apparatus, and their life cycle is coordinated with the maturation cycle of *G. intestinalis* flagella. The orchestration of mitosomal inheritance with the flagellar maturation cycle is mediated by a microtubular connecting fiber, which physically links the privileged mitosomes to both axonemes of the oldest flagella pair and guarantees faithful segregation of the mitosomes into the daughter cells.

**Conclusion:**

Inheritance of privileged *Giardia* mitosomes is coupled to the flagellar maturation cycle. We propose that the flagellar system controls segregation of mitochondrial organelles also in other members of this supergroup (Metamonada) of eukaryotes and perhaps reflects the original strategy of early eukaryotic cells to maintain this key organelle before mitochondrial fusion-fission dynamics cycle as observed in Metazoa was established.

**Supplementary Information:**

The online version contains supplementary material available at 10.1186/s12915-021-01129-7.

## Introduction

Mitochondria are indispensable for producing most cellular ATP and providing other metabolites such as amino acids, lipids, and iron-containing prosthetic groups like heme and iron-sulfur (FeS) clusters [1]. Upon changing cellular conditions, their morphology undergoes constant re-arrangements known as mitochondrial dynamics, through which the cell controls metabolic capacity and the health of its key organelles [2]. Mitochondria either fuse to generate intricate networks throughout the cytoplasm or divide to form discrete organelles which more resemble their bacterial ancestors [3]. At the center of molecular machinery mediating the mitochondrial dynamics are dynamin-related proteins (DRPs) [4], but imperfect mitochondria are removed from the fusion-division cycle via specialized autophagy pathway [5]. Typical animal cells carry one to two thousand mitochondria which are stochastically segregated into two daughter cells during the cell division [6].

Studies on mitochondrial dynamics across different supergroups of eukaryotes showed that the extensive ongoing cycles of division and fusion are rather derived behavior of mitochondria not seen outside animal and fungal cellular models. This specifically concerns mitochondrial fusion which is either absent [7] or rely on unknown molecular machinery [8].

From the evolutionary perspective, it is likely that the early eukaryote contained a single mitochondrion that divided in synchrony with the cell as often observed for the bacterial endosymbionts of current eukaryotes [9]. Some eukaryotes have preserved this original blueprint of a single mitochondrion [10–13] which divides in synchrony with the entire cell. However, only in case of kinetoplastids, the fundamentals of the synchrony were uncovered to some molecular detail [14–18]. Herein, so-called Tripartite Attachment Complex (TAC) physically connects basal body to the mitochondrial (mt) genome organized into a structure known as kinetoplast [17]. Specific TAC components were characterized across the complex from the basal body side (e.g., BBA4) [19], over the mitochondrial membranes (e.g., TAC40, TAC60) [20, 21] to the mitochondrial matrix (TAC102) [22], and their function is to coordinate the segregation of the replicated mt genomes/kinetoplast.

*Giardia intestinalis* is an anaerobic unicellular eukaryote (protist) from the Metamonada group which encompasses only organisms adapted to anoxic environments [23]. Accordingly, their mitochondria have been highly reduced to hydrogenosomes or mitosomes which are still cordoned by a double membrane but lost most of the other mitochondrial attributes such as the genome and respiratory chain [24]. *Giardia* mitosomes are the most reduced form of mitochondria as they are about 50–200 nm in size, do not produce ATP, and harbor only single metabolic pathway of FeS cluster formation [25]. Interphase mitosomes are very steady organelles, which do not fuse. Strikingly, their division seems to occur only during mitosis [26–28]. Of about the total 40 mitosomes per cell, several organelles can be, without exception, found between the two *Giardia* nuclei [25, 29–31]. These observations indicated specific attachment of the mitosomes to the nuclei or basal bodies of eight *Giardia* flagella. However, the molecular and structural basis for the recruitment of the organelles to this particular region of the cell and its actual function remained unknown.

Here, we tried to elucidate the nature of the association and its biological consequences towards the mitosis and cytokinesis of *Giardia.* Regarding the minute character of mitosomes, we used STED and FIB/SEM tomography to demonstrate that mitosomes are, via specialized microtubular fiber, bound to the axonemes of the oldest pair of *Giardia* flagella. We show that mitosomal inheritance is controlled by flagellar maturation and segregation. Comparisons with other Metamonada species suggest that the connecting fiber is a more common structure and perhaps one of the ancestral mechanisms to control mitochondrial segregation. Altogether, we demonstrate that the inheritance of the minimalist and genomeless mitochondrial organelles is under a careful control of the eukaryotic cell.

## Results

### Pre-existing central mitosomes segregate during prophase towards the poles of the mitotic spindle

**Fig. 1:**
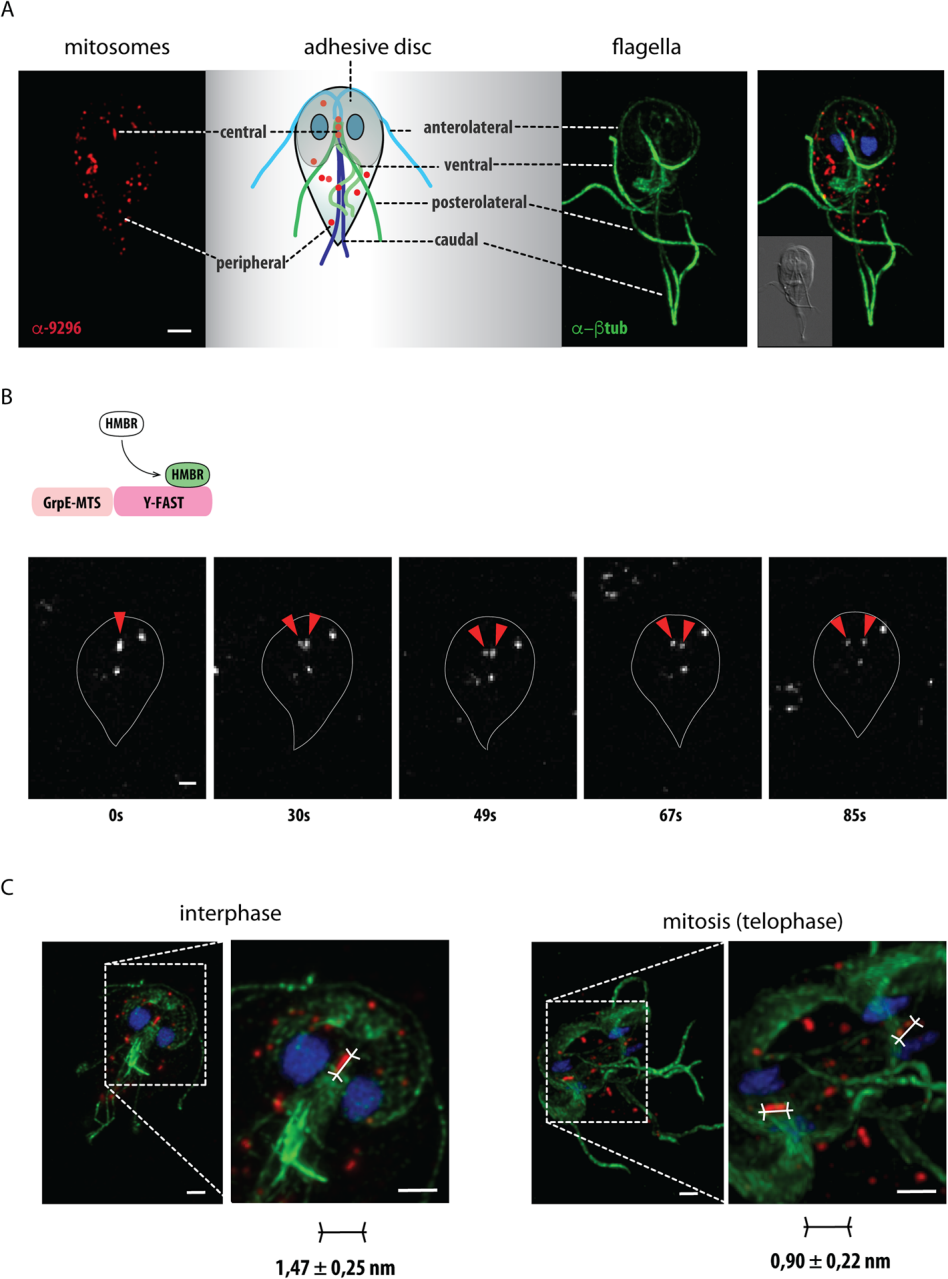
Partitioning of *Giardia* central mitosomes during mitosis. **A** The schematic drawing of *Giardia* trophozoite depicts central and peripheral mitosomes (red). The first are found between two nuclei, where also basal bodies and axonemes of *Giardia* flagella are localized. The latter are scattered across cytoplasm. There are four pairs of flagella called according to their position in the cell as anterolateral (light blue), posterolateral (dark green), ventral (light green), and caudal (dark blue). Additionally, the helical sheet of microtubules forms the adhesive disc of *Giardia* (gray). Immunofluorescence microscopy image of mitosomes labeled by anti- GL50803_9296 antibody (red), anti-β-tubulin. Merged image on the right includes nuclei stained with DAPI. The inlet is DIC image of the cell. **B** *Giardia* expressing the fusion of targeting presequence of mitosomal GrpE and Y-FAST were enriched for mitotic cells using albendazole treatment and incubated with 4-hydroxy-3-methylbenzylidene-rhodanine (HMBR) substrate. The reversible binding of Y-FAST to the fluorogenic substrate induces its green fluorescence (530–540 nm) upon excitation at 470–480 nm. The observed plane was focused on the central mitosomes. The series of images demonstrates fast partitioning of the fluorescence corresponding to central mitosomes. Red arrowheads highlight the position of central mitosomes. **C** The size of the fluorescent signal corresponding to the central mitosomes in the interphase and mitotic cells (the averaged values of 30 cells are shown). The scale bars 2 μm.

All active cells of *Giardia* (trophozoites) contain two different populations of mitosomes, described as central and peripheral, which occur between two *Giardia* nuclei or are distributed all over the cytoplasm, respectively (Fig. 1A) [28, 29, 32]. While the latter predominantly associate with the tubules of the endoplasmic reticulum (ER) [28], the central mitosomes form a short array of several adjacent organelles between two *Giardia* nuclei which stay extremely steady until the initiation of mitosis [25, 31, 33]. Both the central and the peripheral mitosomes seem to be functionally identical as all mitosomal proteins detected by specific antibodies or by overexpression of the tagged proteins localize to both populations. All mitosomes, central and peripheral, divide during mitosis [28]. During early prophase, a rod-like array of mitosomes develops into a transient V-shape assembly which indicates a division or segregation of the organelles towards the opposite sides of the mitotic spindle [28]. To test if two sets of central mitosomes are formed during mitosis, we followed their behavior in live. To this aim, we took advantage of recently developed enzymatic Yellow Fluorescence-Activating and absorption-Shifting (Y-FAST) tag which outperforms GFP and related fluorescent proteins in anoxic environments [34]. The culture of *Giardia* cells expressing the fusion of mitosomal targeting presequence of GrpE with Y-FAST was enriched with the mitotic cells (see the “Methods” section). The cells were mounted to 0.5% agarose and observed in live in the presence of HMBR substrate. Upon focusing on the plane of the central mitosomes, we were able to detect rare events of the partitioning of the fluorescent signal corresponding to central mitosomes (Fig. 1B, Additional file 1: Movie S1). We estimated that the partitioning occurred during a 12-s window. As an alternate approach, we compared the size of the fluorescent signal of the central mitosomes in the fixed interphase cells and mitotic cells undergoing early prophase (Fig. 1C). The calculated values were 1.47 ± 0.25 μm and 0.90 ± 0.22 μm for the interphase and the mitotic cells (each *n* = 50), respectively. This supported the split of the fluorescent signal corresponding to the central mitosomes and further suggested that either the segregation of pre-existing mitosomes or the actual division of the organelles occurs during mitosis.

### Mitosomes associate with the axonemes of *Giardia* flagella

**Fig. 2:**
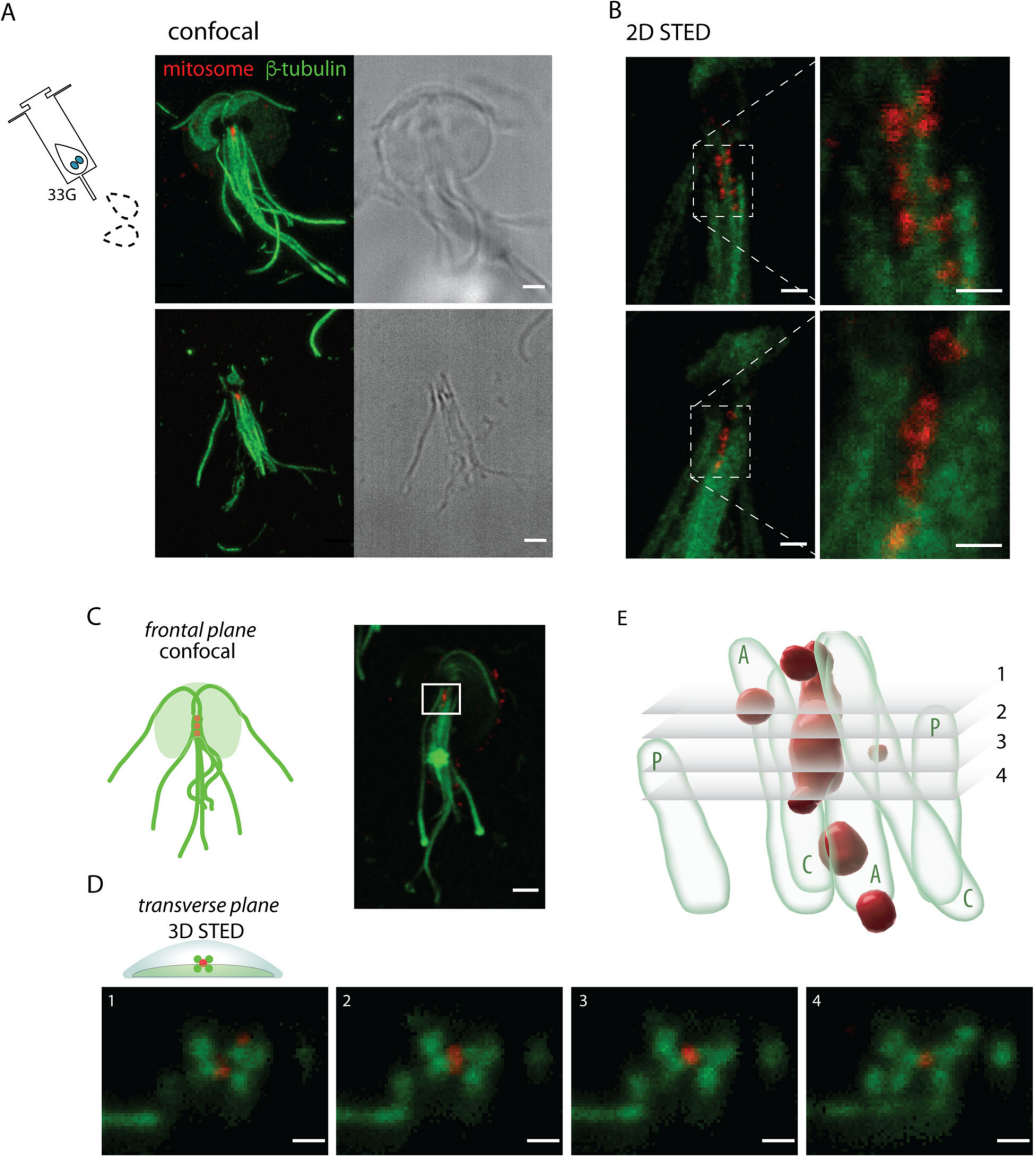
Central mitosomes associate with the flagellar axonemes. **A** *Giardia* cells were gently lysed by the passage through 33G needle, and the ruptured cells were analyzed by immunofluorescence microscopy. The immunolabeling of β-tubulin (green) and mitosomal marker GL50803_9296 (red) revealed that the intact mastigonts bear attached central mitosomes, while the cytoplasmic membrane and rest of cytoplasm was removed during lysis, scale bar 2 μm. Upper and bottom panels represent two examples of the same situation. **B** 2D STED superresolution microscopy of the mastigonts with associated mitosomes (primary antibodies as in **A**), scale bar 0.2 μm (0.1 μm in the insets), 30 nm resolution in XY axes. Upper and bottom panels represent two examples of the same situation. **C** The central mitosomes imaged in the frontal plane, scale bar 2 μm, and in **D** the transverse plane by 3D STED superresolution microscopy (primary antibodies as in **A**). Four optical sections are shown, scale bar 0.4 μm; the resolution was 100 nm in *Z*-axis and 120 nm in XY plane. **E** The reconstruction of 3D STED by Imaris illustrates the position of mitosomes (red) among eight axonemes (green), sections shown in **D** are indicated. C, A, P – caudal, anterolateral and posterolateral flagella, respectively.


The stable position of the central mitosomes between two *Giardia* nuclei together with the segregation of two mitosome populations during the onset of mitosis indicated some sort of physical association with the intracytoplasmic axonemes or basal bodies of flagella. *Giardia* basal bodies were suggested to act as microtubule organizing centers (MTOCs) of the mitotic spindle as they become a part of the spindle poles during the re-orientation of two nuclei [35–37]. To experimentally test the presence of the physical linkage between mitosomes and flagellar cytoskeleton, the cells were gently lysed by the passage through a hypodermic 33G needle (see the “Methods” section). The lysed cells were fixed and mitosomes and microtubules labeled by specific antibodies (Fig. 2A). Indeed, the lysed cells showed an intact association of the central mitosomes with the axonemes, even when the rest of the cell cytoplasm was removed during the sample preparation. In contrast, there were no peripheral mitosomes observed. Furthermore, the samples were prepared for 2D stimulated emission depletion (STED) microscopy (Fig. 2B) which revealed that in the partially disturbed bundle of the axonemes, mitosomes can be found associated only with some of the axonemes. Alternate 3D-DyMIN STED approach [38] was used to image the region on more intact samples from the transverse plane (Fig. 2C, D). Here, the central mitosomes were found surrounded by axonemes, right at the center of their bundle (Fig. 2E, Additional file 2: Movie S2 and Additional file 3: Movie S3). These results suggested that an unknown connector between the axonemes and the central mitosomes is responsible for the organelle segregation during cell division.

**Additional file 3: Movie S3.** Movie of Imaris reconstruction of central mitosomes built on 3D STED.


### Imaging of the mitosomes using FIB/SEM tomography

**Fig. 3:**
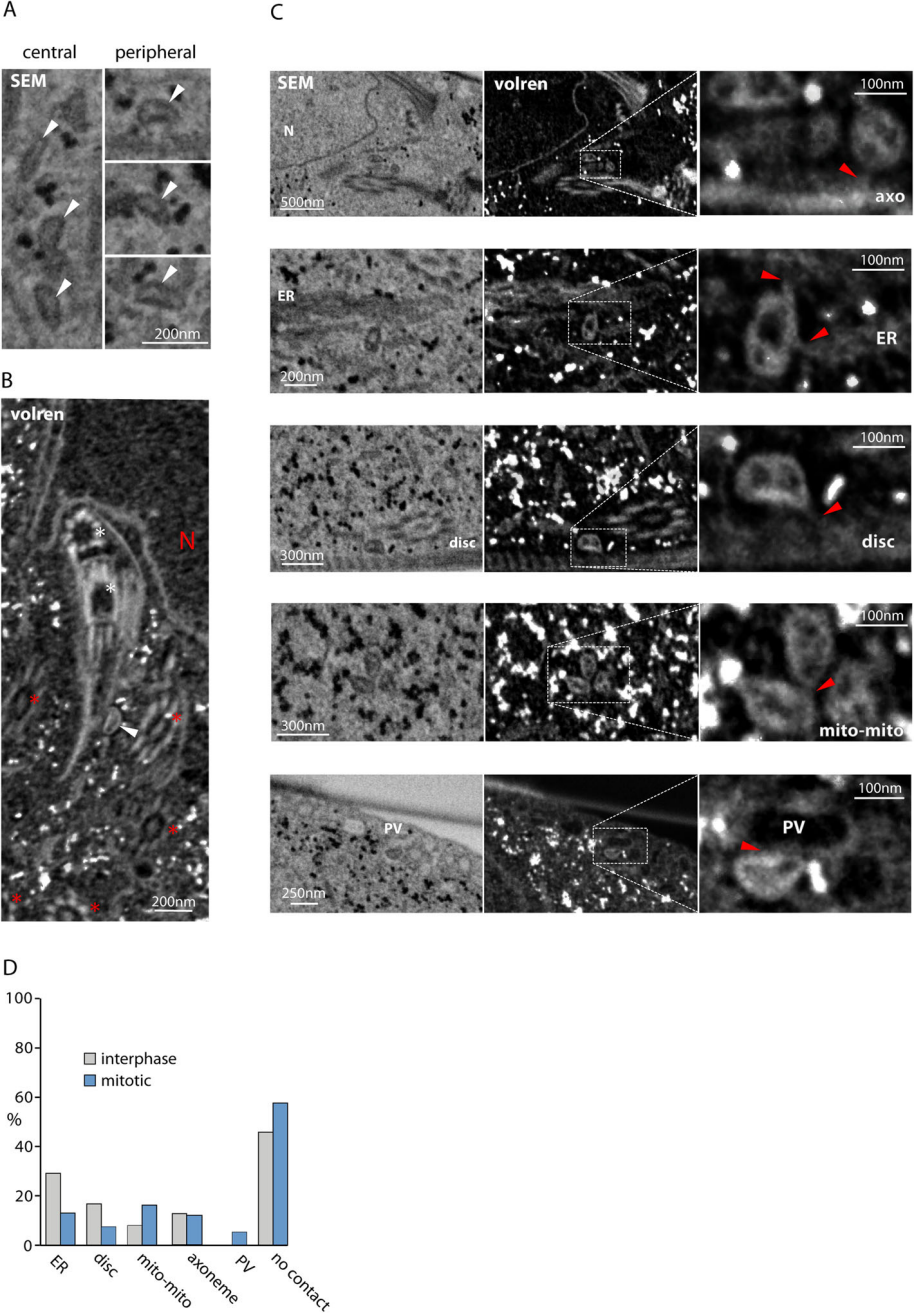
FIB/SEM imaging of mitosomes reveals contacts with other cellular compartments. **A** The SEM imaging was optimized to enable visualization of the mitosomes. The examples of central and peripheral organelles are shown. They all share asymmetric, semi-oval shape with one flattened side of the organelle. **B** The volume rendering (volren) of several SEM layers increases the resolution of the imaging of the cell interior. N-nucleus, white arrowhead-mitosome, red star-axoneme, white star-basal body. **C** The mitosomes were found to associate with several cellular compartments, i.e., the axonemes (AXO), endoplasmic reticulum (ER), adhesive disc (disc), other mitosomes (mito-mito), and the peripheral vacuoles (PV). The red arrowheads point to visible connection. **D** The distribution of the contacts of mitosomes with other cellular compartments in the interphase and the mitotic *Giardia* (*n* = 48 and *n* = 106 organelles, respectively)

**Fig. 4:**
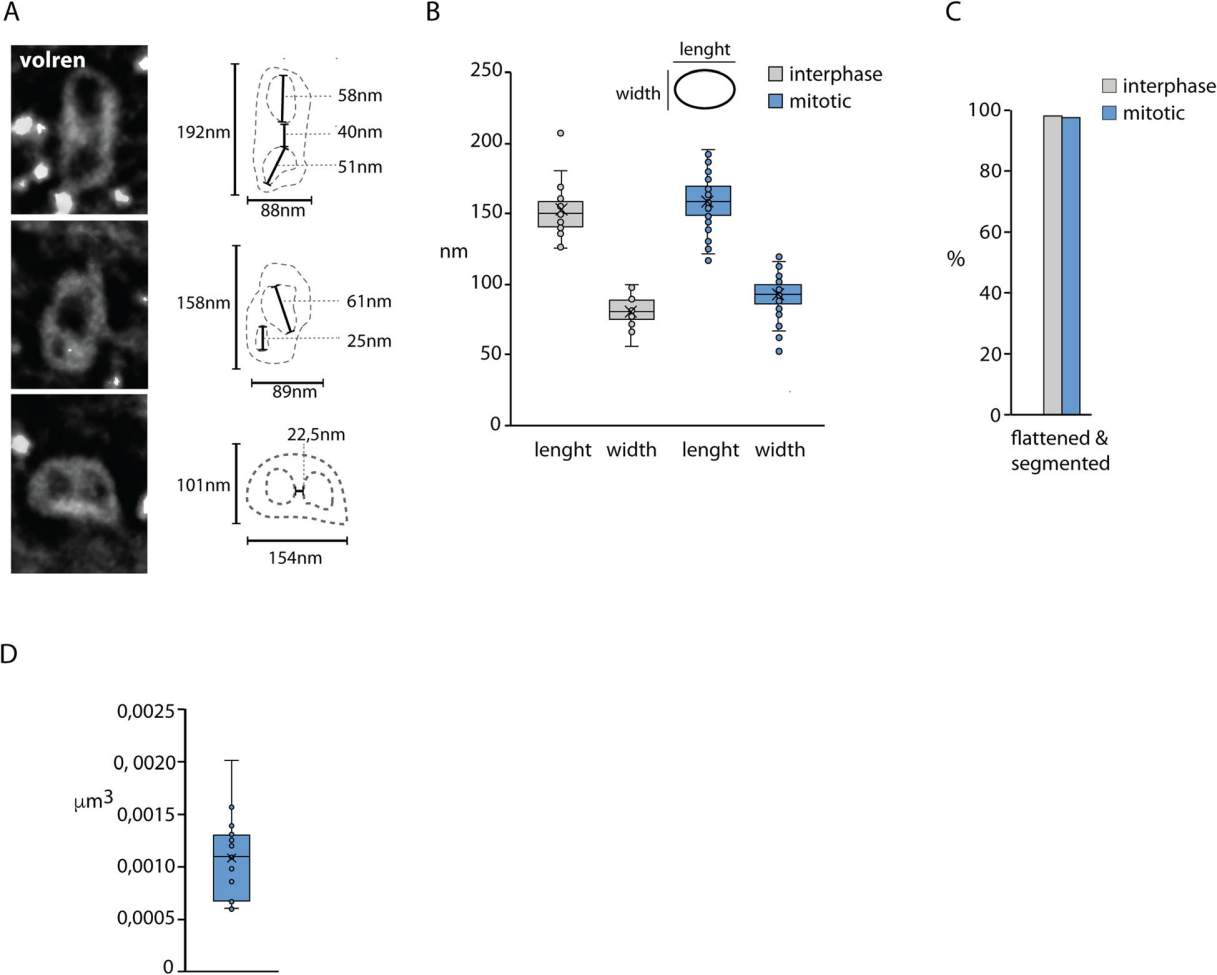
The mitosomes are segmented and share the asymmetric shape with one flattened side. **A** Mitosomes show internal compartmentalization. The examples of different size and architecture of the organelles are shown. **B** Statistics of the mitosomal dimensions show conserved organelle shape (*n* = 30 and *n* = 70 organelles in the interphase and mitotic cells, respectively). **C** Almost all mitosomes have flattened shape and segmented interior (*n* = 48 and *n* = 106 organelles in the interphase and mitotic cells, respectively). **D** The estimated mitosomal volume (*n* = 17 organelles)

While the confocal and superresolution microscopy provided experimental support for the presence of a molecular bridge or a cytoskeletal connector between the central mitosomes and the axonemes, the nature and the architecture of the connection remained entirely unknown. Hence, the high-resolution imaging of the entire *Giardia* cells by electron microscopy seemed essential to obtain such structural details. Specifically, focused ion beam scanning electron microscopy (FIB/SEM) was employed as it enables to reconstruct spatial arrangement of cell structures upon rendering the information of sequential data sets. To this aim, *Giardia* cells were flat embedded (Additional file 4: Fig. S1) [39] and, using the correlative approach, the interphase and mitotic cells were selected for FIB/SEM analysis (Additional file 4: Fig. S1, see the “Methods” section). The cells were processed by customized rOTO protocol and analyzed by fine milling (voxel size 3 × 3 × 8 nm) [39, 40] (Fig. 3A, B, Additional file 5: Fig. S2). The 3D reconstruction of the mitosomes throughout the entire cell volume showed that in addition to the axonemes, approximately one half of the mitosomes (central and peripheral) is in connection to other cellular structures such as the ER, the adhesive disc, and lysosome-like compartments known as peripheral vacuoles [41–43] (Fig. 3C, D). Regularly, there was only one contact with other compartments detected per mitosome. Finally, clusters of several (peripheral) mitosomes could be found in the cytosol (Fig. 3C). In general, the association of mitosomes with cellular compartments was comparable between interphase and mitotic cells (Fig. 3D). The inspection of the mitosomal interior revealed further compartmentalization/segmentation (Fig. 4A, C), suggesting that the two mitosomal membranes do not align entirely and the inner membrane establishes tiny mitosomal subcompartments. The previous EM images of the mitosomes revealed non-spherical prolonged shape of the organelles [25, 32]. In addition, we noticed that mitosomes share one flattened side, i.e., contain flat regions of double membrane (Fig. 3A–C, Fig. 4C). This shape was common to mitosomes from all parts of the cell with different orientation to the cell axis concluding that the flattening is an intrinsic property of the organelles not caused by sample preparation or neighboring cell structures.

In order to describe this shape quantitatively, the longest dimension (length) and the dimension perpendicular to it (width) were measured and plotted (Fig. 4B). The both dimensions averaging around 150 nm and 75 nm, respectively, were found to be very conserved across mitosomes, hence suggesting that the mitosomal size and shape are defined by the intrinsic protein and lipid composition. In addition, vast majority of the organelles contained membrane indentations forming possible additional subcompartments (Fig. 4C, E). Finally, we used 3D reconstructions of 17 mitosomes to estimate that the average mitosomal volume is around 0.0011μm^3^ (Fig. 4D). In the context of the estimated volume of the entire *Giardia* trophozoite to be 440 μm^3^, all (forty) mitosomes represent only a fraction of 0.00025% of the whole cell volume.

### Central mitosomes are connected to only the eldest (caudal) pair of flagella via a microtubular connector

**Fig. 5:**
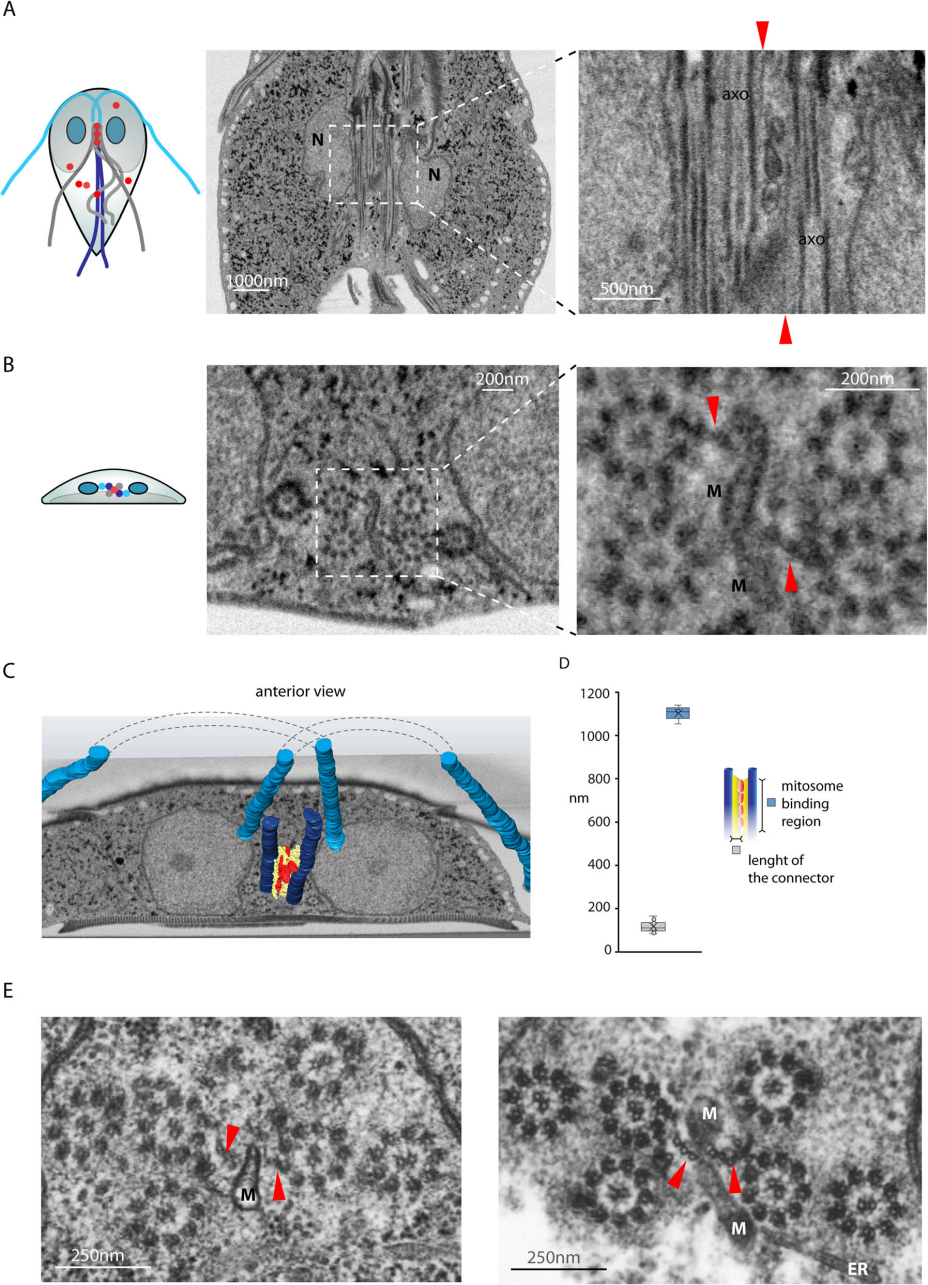
FIB/SEM analysis reveals connecting microtubular fiber between central mitosomes and the axonemes of caudal flagella. **A** The frontal plane image of the central mitosomes of interphase *Giardia* between the longitudinally sectioned axonemes as reconstructed from the transversal FIB/SEM images, AXO—axoneme, red arrowhead—microtubular fiber, N—nuclei. **B** The illustrative transversal section shows two fibers (red arrowheads) connecting central mitosomes and axonemes of caudal flagella. M—mitosomes. **C** The reconstruction of the FIB/SEM analysis depicting the overall architecture of the microtubular fiber (yellow) between central mitosomes (red) and axonemes of caudal flagella (dark blue). Axonemes of anterolateral flagella are shown in light blue, N-nuclei. **D** The dimensions of the connecting fiber (*n* = 15 and *n* = 3 for the length and of the connector and the binding region, respectively). **E** Transmission electron micrographs show the detailed structure of the connecting microtubular fibers, which each consists of 3 to 4 microtubules. M-mitosomes

The examination of the region of interest between two *Giardia* nuclei by FIB/SEM tomography was facilitated by the characteristic pattern of axonemes of the eight *Giardia* flagella (Fig. 5A). There are four distinct pairs of flagella named according to positions at which they emerge from the cell as ventral, posterolateral, anterolateral and caudal (Fig. 1A). Importantly, during each cellular division, the flagella undergo a flagellar maturation cycle in which two caudal flagella represent the eldest pair of the flagellar cycle [36].

In the FIB/SEM micrographs of the region of tightly packed axonemes between *Giardia* nuclei, a population of several central mitosomes could be seen, showing a typical array of the organelles as observed by fluorescence microscopy (Fig. 5A). The number of central mitosomes was slightly variable with the average number to be eight in the interphase cells (*n* = 5). Detailed analysis of the individual micrographs revealed a direct linear connection between each of the central mitosomes and always one of the two neighboring axonemes (Fig. 5A, B). Interestingly, only two of eight axonemes were involved in mitosome binding and the overall reconstruction revealed that these correspond to the pair of caudal flagella (Fig. 5C). However, it could not be resolved, if a specific microtubule doublet is involved in the binding. The length of the connector (distance between the mitosome and the axoneme) was estimated to be around 90 nm (Fig. 5D), while the actual mitosome-binding region of the connector in the anteroposterior axis was approx. 1.1 μm (Fig. 5D. The connector itself accompanied the whole length of the intracytoplasmatic portion of caudal flagella (Fig. 5C).

To get higher resolution of the connector, *Giardia* cells were examined by transmission electron microscopy (TEM). While TEM was not optimal for mitosome investigation, it nicely exposed the structural features of the connector as a sheet of microtubules—a microtubular fiber (Fig. 5E). Hence, we could conclude that the central mitosomes are specifically linked via microtubular fiber to the axonemes of the caudal flagella. Further microscopic examination revealed that the mitosomal connector is integrated into the upper part of the so-called funis system of *Giardia*, which represents the cytoplasmic microtubular skeleton alongside the two caudal flagella (Additional file 6: Fig. S3) [44, 45].

### Coordination of mitosomal inheritance and the flagellar cycle

The data presented above demonstrated that mitosomes segregate during mitosis to the opposite ends of the mitotic spindle probably via the link to caudal flagella. This has raised a fundamental question on the coordination of mitochondrial inheritance and the flagellar cycle, during which a new caudal flagellum is established by the transformation of one (mitosome-free) anterolateral flagellum. Of particular importance was (i) at what stage the connecting microtubular fiber develops on the axoneme of new caudal flagellum, (ii) when the central mitosomes are acquired, and (iii) whether the new central mitosomes are derived from the pre-existing organelles linked to the old caudal flagellum.

**Fig. 6:**
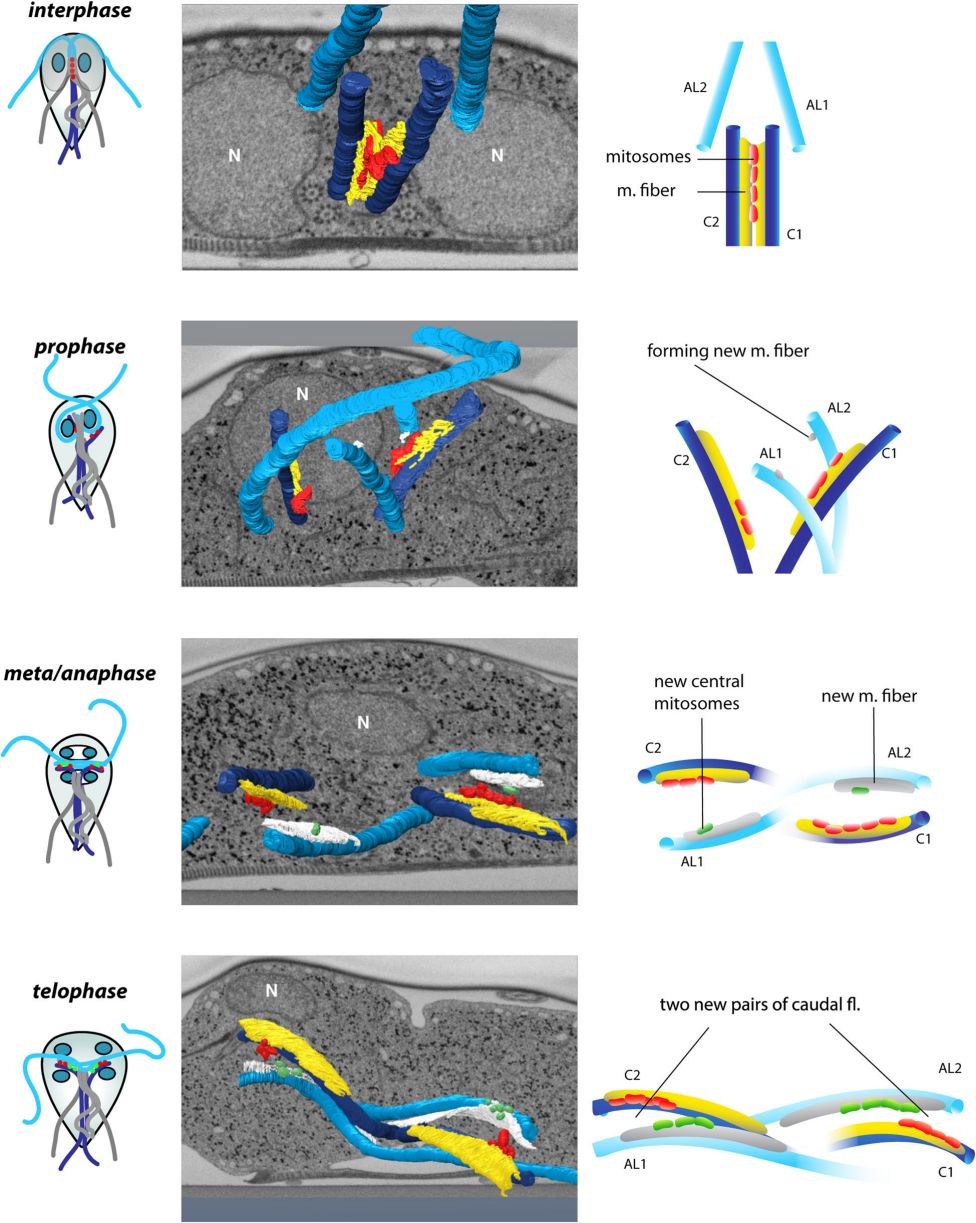
The orchestrated mitosomal segregation and flagellar maturation during mitosis. 3D reconstructions of mitosomal and flagellar dynamics during mitosis shows (i) separation of the caudal flagella with the connected mitosomes during prophase. Note also the formation of the new connecting microtubular fiber on both anterolateral flagella. (ii) During meta/anaphase the mitosomes become associated with the new fiber on the former anterolateral flagella. (iii) During telophase, two new pairs of caudal flagella (the coloring according to the original interphase cell) are formed with more mitosomes (green) connected to the new caudal flagellum. AL1, AL2—anterolateral flagella; C1, C2—caudal flagella

To get appropriate answers, different stages of mitotic *Giardia* were examined by FIB/SEM tomography. The flagellar re-arrangement starts at prophase when basal bodies/axonemes of caudal flagella deflect to the left and right side to set up the future sites of daughter cells morphogenesis. This is accompanied by a profound reorientation of anterolateral flagella axonemes (Fig. 6), which migrate to the opposite direction and each join the other/one caudal flagellum at the opposite side of the cell [36]. At that time the central mitosomes split into two populations, each alongside one caudal flagellum (Fig. 6). At the re-oriented anterolateral flagellum, a new tiny connecting fiber (150–300 nm in length) is being formed, however, at this stage, without attached mitosomes (Fig. 6).

**Fig. 7:**
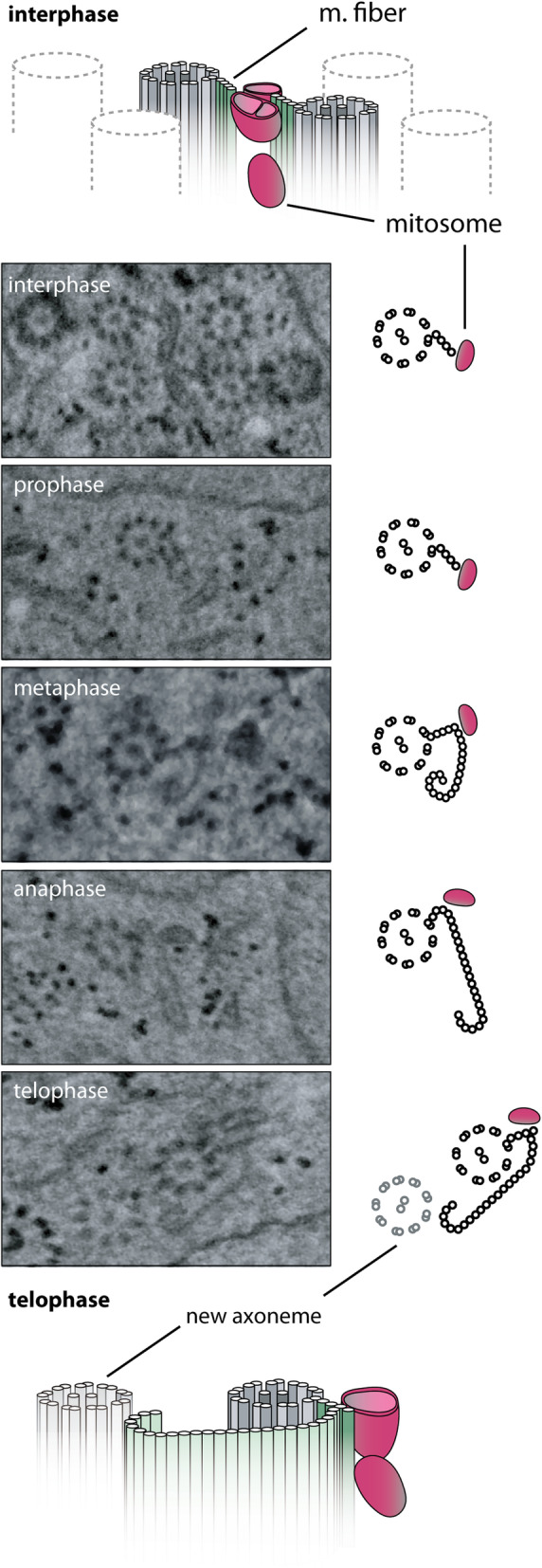
The development of connecting microtubular fiber during mitosis. During the mitotic stages, the microtubular fiber extends and points towards the newly built axoneme. New extended vaulted microtubular part grows towards the newly assembled flagellar axonemes of daughter ventral and posterolateral flagella (light gray axoneme) and the fiber curls around a single microtubule (red)

During metaphase and anaphase, the fibers at caudal flagella acquire a new extended vaulted microtubular part with the distal curled end (Fig. 7). The accompanying mitosomes, however, remain associated at the end of the linear part of the fiber as seen in interphase and prophase. The number of mitosomes attached to caudal flagellum varies at this stage from 1 up to 6 with typically 4 organelles (*n* = 6). The re-oriented anterolateral flagellum which joined the caudal flagellum transforms into a new caudal flagellum. The transformation is characterized by an apparent growth of the accompanying microtubular fiber, to which mitosomes are recruited (Fig. 6). At this stage, the shape of the fiber fully resembles the one of the old caudal flagellum.

During telophase, both old and new caudal (i.e., transformed anterolateral) axonemes have already reached their positions at the anterior of the future daughter cell. The elongation of the microtubular fiber reaches up to 3 μm in length. Interestingly, the vaulted extension of the fiber is oriented towards the newly assembled flagellar axonemes of daughter ventral and posterolateral flagella and may provide morphogenetic signals for oriented intracytoplasmic flagellar growth (Fig. 7). Strikingly, at this stage, the mitosomal populations at the old and new caudal flagella are of similar size (4–6 mitosomes), strongly indicating that the new caudal flagellum might recruit its mitosomes from the surrounding peripheral organelles (Additional file 7: Fig. S4). Finally, the closer pairing of the axonemes of two caudal flagella guides the two mitosome populations together (Fig. 6, Additional file 8: Movie S4). The two microtubular fibers accompanying caudal flagella become each a ventral and a dorsal part, respectively of the daughter cell funis (Additional file 5: Fig. S2).


**Additional file 8: Movie S4.** Animation of 3D reconstruction. Caudal flagella in dark blue, anterolateral flagella in light blue. The old and new connecting fibrils shown in yellow and white, respectively. Central mitosomes shown in red.


### Segregation of mitosomes occurs also when mitosis is aborted

**Fig. 8:**
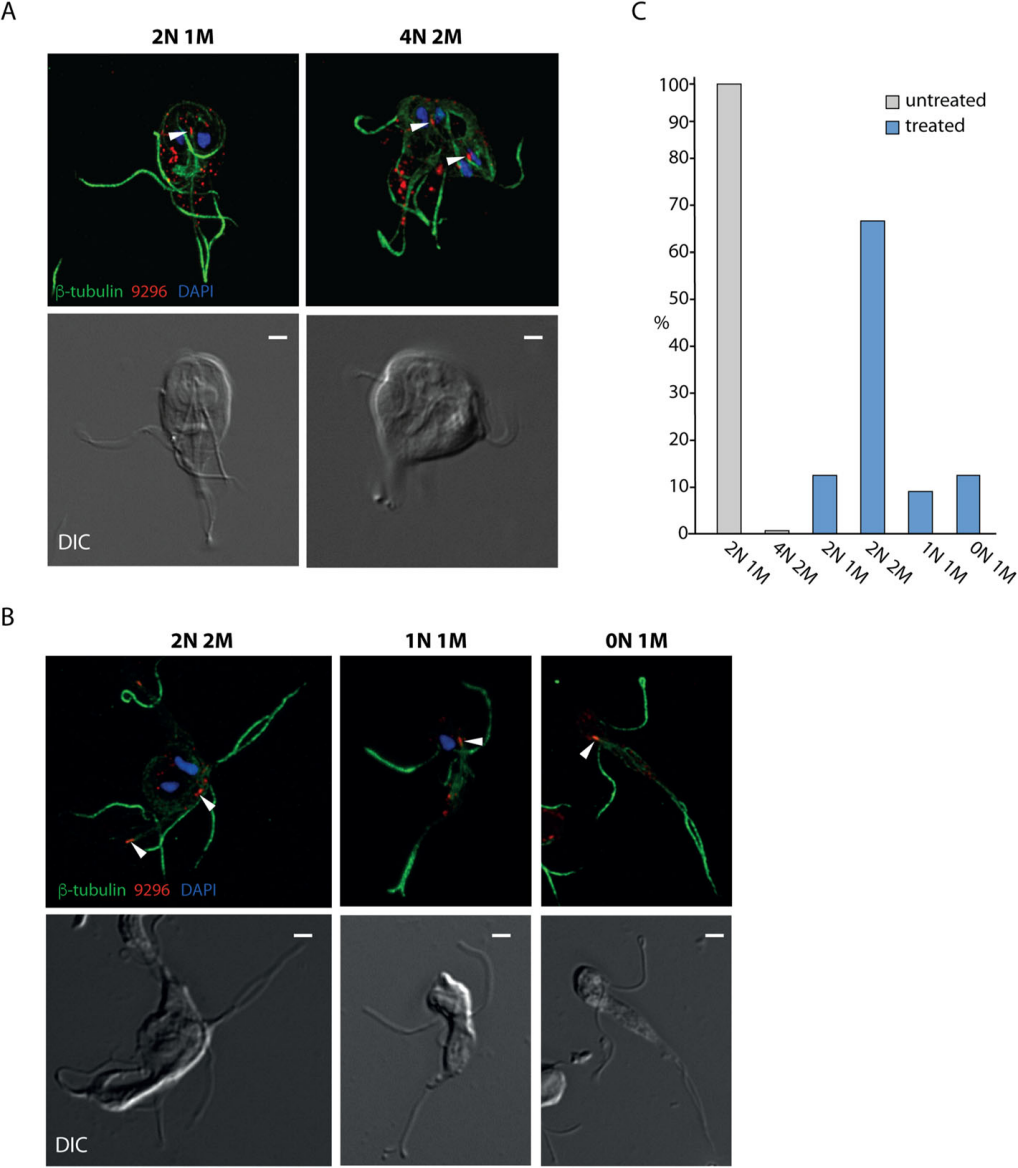
Segregation of mitosomes occurs also when mitosis is impaired. The inhibition of the microtubule assembly results into the formation of aberrant cells with impaired nuclear segregation. **A** Vast majority of cells in untreated cell population are in the interphase stage containing two nuclei (N) and one flagellar apparatus (M) (2N1M). Less than 1% of cells undergo mitosis that ends with a cell carrying 4 nuclei and 2 flagellar apparatuses (4N2M). **B** Four cell types can be detected in the cell population classified according to the number of the nuclei and the flagellar apparatuses. The cell type 2N2M is pre-cytokinesis; the other cell types represent cells which underwent cytokinesis. Examples of the aberrant morphologies are shown. Immunolabeling by anti-β-tubulin antibody (green), mitosomes by anti-GL50803_9296 antibody (red). Nuclei are stained with DAPI (blue). The central mitosomes highlighted by white arrowheads. **C** The quantification of morphologies in albendazole treated (*n* = 334 cells) and untreated (*n* = 350 cells) populations

The discovery of the tight association of *Giardia* mitosomes with the flagella and the orchestration of mitosome segregation during mitosis prompted us to investigate the impact of mitosis on mitosome dynamics. *Giardia* cells were incubated with albendazole which is a potent inhibitor of newly polymerized microtubules. The affected *Giardia* cells retain functional microtubular structures but are unable to assemble new microtubules such as those composing the mitotic spindle [46]. Cells visually undergoing cytokinesis were analyzed by fluorescence microscopy and compared to untreated control (Fig. 8A, B). Immunolabeling of mitosomes and microtubules along with DAPI staining showed several distinguishable phenotypes (Fig. 8) [46], which were classified according to the number of nuclei (N) and the number of the flagellar apparatuses with the associated structures (M). While the untreated population contained almost exclusively interphase stage trophozoites with two nuclei and one flagellar apparatus (2N1M) and a small number (less than 1%) of mitotic cells (4N2M) (Fig. 8A, C), four different phenotypes could be distinguished in the albendazole treated cell culture (Fig. 8B). The most cells had two (undivided) nuclei and two separated flagellar apparatuses (2N2M), indicating that they did not complete cytokinesis (Fig. 8B, C). Three other phenotypes included cells with 0N1M and 2N1M arrangements and the least abundant 1N1M arrangement (Fig. 8B, C). There was also a small population of unaffected interphase cells with two nuclei and one flagellar apparatus (2N1M). In all cases, regardless of the number of cell nuclei, each flagellar apparatus co-localized with the fluorescent signal corresponding to the central mitosomes (Fig. 8A, B). The inhibition of mitosis hence did not impact the segregation of the central mitosomes, which remained associated with the caudal flagella by the existing microtubules. This result showed that it is exclusively the flagella and not the nuclei that have control over the segregation of central mitosomes to the daughter cells.

## Discussion

The eukaryotic cell requires its mitochondria to be faithfully inherited by the daughter cells during the cell division. Being semiautonomous organelles with their genome and translation machinery, mitochondria propagate only from the pre-existing organelles [2, 47, 48]. Our current understanding of the history of the eukaryotic cell infers that its formation coincided with the origin of its mitochondrion [24], and hence, the mitochondrial inheritance has been an integral part of eukaryotic cellular functions from its early days.

Mitochondria of Opisthokonta (animals and fungi) have served as the principal models for studying mitochondrial dynamics and inheritance, e.g., [49–51]. Moreover, multiple health and developmental problems have been related to the erroneous dynamics of human mitochondria [51–53]. Herein, hundreds of mitochondria constantly fuse and divide to control the quality of the organelle and its genome with the assistance of the ER [54] and actin cytoskeleton [55]. The inheritance of the dynamic animal mitochondrial network thus occurs rather stochastically, even though there seems to be also cell cycle-dependent aspects of mitochondrial dynamics [6, 56, 57].

In contrast, there are large groups of eukaryotes like Kinetoplastida or Apicomplexa, which entirely harnessed the mitochondrial inheritance with their cell cycle [14–17, 58–60]. These unicellular eukaryotes carry only a single mitochondrion, which thus must be carefully propagated to the daughter cells. Kinetoplastida have evolved a structure called tripartite attachment complex (TAC), which physically connects the mitochondrial genome with the basal body of its flagellum [17]. The segregation of the basal body is thus necessary for the partitioning of the duplicated mitochondrial genome (kinetoplast), which is quickly followed by nuclear division and cytokinesis [14]. In addition, a prominent microtubule quartet extending along the length of the cell plays a key role in the coordinated biogenesis of different organelles during the cell cycle [61].

Apicomplexa evolved schizogony, a synchronized cell division resembling intracellular budding, during which mitochondrial filaments enter into the emerging daughter cells [60, 62].

**Fig. 9:**
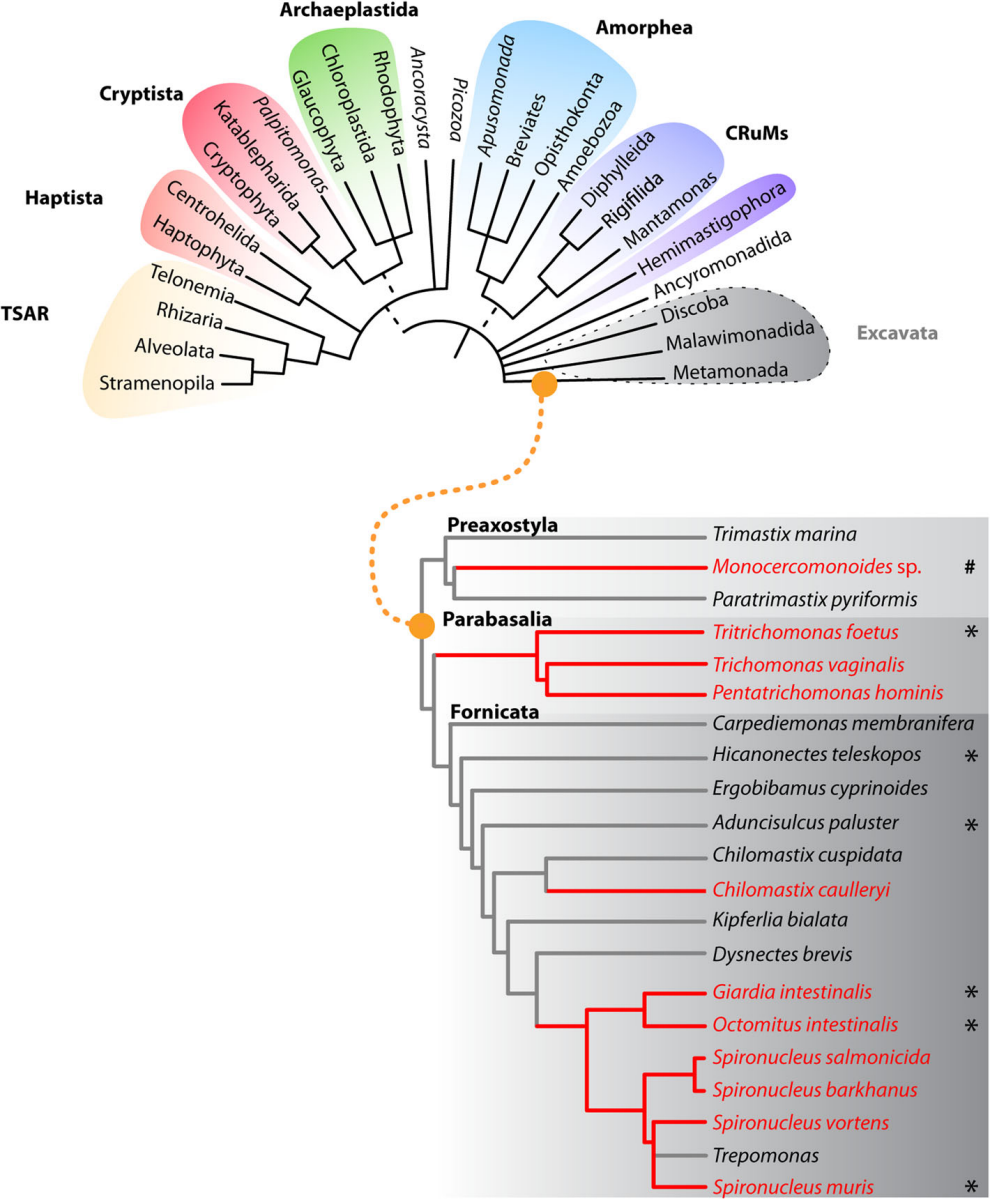
Connecting the mitochondrial organelles to the flagellum may be an ancestral strategy of controlling the mitochondrial inheritance in Metamonada. The schematic tree of eukaryotic supergroups drawn according to [63] shows the position of Metamonada and within recently questioned Excavata supergroup. Bellow, a subtree of selected Metamonada species according to [64, 65]—the parasitic lineages are shown in red. Highlighted is the presence of the microtubular fiber or analogous structures (*) *T. foetus* [66], *H. teleskopos* [67], *A. paluster* [68], *O. intestinalis* [69], and *S. muris* [70]. Note the secondary absence of mitochondrial organelle in *Monocercomonoides* sp. (#)

Eukaryotes from the supergroup of Metamonada (Fig. 9) [63] are all intriguingly adapted to inhabit anoxic or low oxygen environments. This has dramatically impacted the structure and function of their mitochondria. They all are genomeless and lack many mitochondrial functions including the respiratory chain and the coupled ATP synthesis. Due to their variability, these organelles are often referred to as mitochondria-related organelles (MROs) [71–74]. Interestingly, MROs are always present in large numbers (in tens to hundreds), and concerning their dynamics, they are very stable during the cell cycle [7]. Yet, virtually nothing is known about how the organisms control their inheritance. Moreover, the genomeless organelles could in principle assemble de novo like the Golgi complex or the peroxisomes, e.g., [75].

We have thus studied the inheritance of mitosomes of *Giardia*, which are one of the simplest and smallest MROs known to date [25]. Mitosomes carry out only a single metabolic role in the formation of iron-sulfur clusters via the mitochondrial ISC pathway [25, 76]. Other metabolic hallmarks of mitochondria have been lost from mitosomes as expendable. However, despite their minimalism, mitosomes are still bounded by two membranes, which carry TOM and TIM complexes to import proteins from the cytosol [30, 31, 77]. Our data show that despite fulfilling the only fraction of the original mitochondrial functions, *Giardia* applies control over their inheritance. It does so by connecting a small subset of mitosomes to flagellar axonemes and specifically to those of the caudal flagella [36, 78]. From the perspective of the flagellar maturation cycle of *Giardia*, the caudal flagella represent the privileged flagellar pair. They are the eldest of four flagellar pairs, in which the left caudal flagellum (in dorsal view) being at the definitive position is considered as truly mature [36]. It serves as the organizing center for the assembly of the adhesive disc [79], which is resorbed during mitosis and re-built in daughter cells upon cytokinesis [80, 81] and for the initiation of the cytokinesis [82].

The examination of *Giardia* mitotic stages revealed that flagellar maturation involves the formation of mitosome-connecting microtubular fiber on the intracytoplasmic axoneme of the newly establishing (formerly anterolateral) caudal flagellum. During mitosis, the fiber rapidly elongates and accompanies the neighboring newly formed axoneme of ventral and posterolateral flagella. Hence, it is possible that the fiber also coordinates the architecture of daughter mastigonts before cytokinesis. The integration of mitosomal and flagellar inheritance was further supported by the experiment on albendazole-treated cells which were not able to undergo karyokinesis but proceeded into divided mastigonts and the central mitosomes.

At what stage do the central mitosomes associate with the newly formed fiber? Our observations strongly indicated that the central mitosomes become “central” only via the association with the connecting fiber as they were never found at this region of the cell just alone. It is noteworthy that also no mitosomal protein has been found exclusively in the central mitosomes [83]. Moreover, according to the most parsimonious scenario, new central mitosomes should be derived from the pre-existing central organelles, when the old caudal axoneme meets the new one. However, surprisingly, we observed mitosomes associated with the yet forming lamella on newly establishing caudal flagellum, before the full establishment of the caudal pair. This indicates that peripheral mitosomes might be able to “hop on” the microtubular fiber at the new caudal flagellum. This view is supported by observations of metaphase/anaphase cells with the already formed fiber at anterolateral flagella, however yet without mitosomes.

The inspection of the fiber beyond the region of the central mitosomes showed that it is likely a part of both the old and of the newly forming microtubular system of *Giardia* and other diplomonads known as funis, possibly R2 root in nomenclature of eukaryotic cytoskeleton [68], which runs in *Giardia* to the posterior end of the cell perhaps to anchor and relax the movement of the eight flagella [45, 69, 84].

To understand whether the microtubular fiber connecting MROs and the axonemes is a more universal cellular feature, we searched the available data on other Metamonada species outside the *Giardia* genus (Fig. 9). Strikingly, highly similar microtubular structure connecting MROs with the axoneme could be observed in the electron micrographs of *Aduncisulcus palustris* [68] and *Hicanonectes teleskopos* [67] which are free-living protists belonging to *Carpediemonas*-like organisms (CLOs) [85]. Together with Diplomonada where *Giardia* belongs, they constitute a larger Fornicata group within Metamonada [23]. Analogously, more distant eukaryotes from Parabasalia group like *Tritrichomonas foetus* are known to associate some of their MROs (hydrogenosomes) with the sheet of microtubules known as axostyle [66]. Hence, it is tempting to speculate that Metamonada at least partially control the inheritance of their genomeless MROs via the association with the stable microtubular cytoskeleton.

Unexpectedly, *Giardia* mitosomes were found to accommodate conserved asymmetric shape. The original discovery of *Giardia* mitosomes already showed the elongated central mitosomes [25]. While this could be explained as a shape imposed on the mitosomes by the neighboring axonemes, our FIB/SEM analysis has revealed that such morphology is also common to the peripheral organelles across the cytoplasm. The vast majority of mitosomes exhibit “bun” or a “bean” shape with opposite oval and flat sides (Fig. 4E). What may be behind such shape? It is well known that phospholipid bilayers spontaneously form spherical vesicles like many endomembrane vesicles or even some double membrane bounded MROs such as mitosomes of *Entamoeba histolytica* [86] or hydrogenosomes of *Trichomonas vaginalis* [87]*.* Other than the spherical shape of the membrane-bounded compartments thus reflects the presence of membrane proteins which affect the membrane flexibility [88]. In case of mitochondria, these can either be proteins that are specifically dedicated to membrane bending and folding such as OPA1/Mgm1 [89], proteins of MICOS components [90] or membrane proteins, whose primary role is unrelated to the compartment morphology, but their sole presence affects the membrane properties such as ATP synthase [91]. So far, no homologs of the actual mitochondrial morphology effectors have been identified in the *Giardia* genome, which could specifically explain the prolonged shape with flat double membrane arrangement. Thus, we can hypothesize that it is perhaps the different content of membrane proteins such as TOM and TIM complexes or so far unknown transport machinery, which stiffen the mitosomal membranes at certain regions. This internal organization occurs in one of the smallest mitochondrial compartments. Our measurements estimated that an average mitosome occupies 0.001 μm^3^ and the whole population of the mitosomes takes up only 0.01% of the cell volume. This is in striking contrast to “classical” aerobic mitochondria of yeast or mammalian tissues which occupy 7% or even 35% of the cell volume, respectively [92–94], and nicely illustrates the overall reduction of the mitosomes and their relative contribution to cellular metabolism. However, despite being such minute compartments, they are carefully watched over by *Giardia.*

## Conclusions

Our work demonstrates a unique type of mitochondrial inheritance which evolved in the anaerobic eukaryotes carrying highly reduced genomeless mitochondrial organelles. While we were able to describe the mechanism underlying the partitioning of the central mitosomes of *Giardia*, this work has raised other exciting questions on the relationship between the central and peripheral mitosomes and, more generally, on the evolutionary importance of the mitochondrion-flagellum connection. Does it represent the ancestral arrangement of the eukaryotic cell or rather a derived trait of the Metamonada group of protists? These questions will hopefully be answered in future studies.

## Methods

### *Giardia intestinalis* cultivation

*G. intestinalis* cells (strains WB, ATCC 30957 and HP-1- Prague line of Portland-1 isolate ATCC 3088) were cultured in TYI-S-33 medium supplemented with 10% heat-inactivated adult bovine serum (Sigma-Aldrich), 0.1% bovine bile, and appropriate antibiotics at 37 °C [95]. The enrichment of mitotic cells was done according to [36]. Trophozoites from late log phase were incubated in growth medium supplemented with 100 ng/ml of albendazole (Sigma-Aldrich) for 6 h at 37 °C [36]. After the incubation, the albendazole-affected detached cells were discarded, and the unaffected adherent pre-mitotic cells were washed twice with pre-warmed fresh drug-free medium and then detached from the tube by cooling on ice for 10 min. The cells were then allowed to proliferate on slides in the drug-free conditions for 9–20 min and fixed by ice-cold methanol (see below) for fluorescence microscopy or by 2.5% glutaraldehyde (see below) for electron microscopy. For the analysis of cells with blocked mitosis, the albendazole-affected (detached) cells were fixed by 1% formaldehyde (see below).

### Plasmid construction and cloning

The first 300 base pairs of the gene GL50803_1376 were amplified from *Giardia* genomic DNA using specific primers: forward 5′-CATGCATATGGCGCTTTCTGCACTT-3′ and reverse 5′-CATGACGCGTGATCTCGTGGAGATGTTT-3′ containing NdeI and MluI restriction sites. After cleavage by NdeI and MluI restriction enzymes, the fragment was cloned into NdeI/MluI-linearized pTG plasmid [96]. The gene encoding Y-FAST protein was amplified from pAG87 plasmid (a kind gift from prof. Gautier, Sorbonne University, France) by forward 5′-CATG*ACGCGT*ATGGAACATGTTGCC-3′ and reverse 5′-CATG*GGGCCC*TTATACCCTTTTGACAAACAC-3′ primers containing MluI and ApaI restriction sites, respectively. After cleavage with MluI and ApaI restriction enzymes, the fragment was cloned into MluI/ApaI-linearized pTG plasmid containing GL50803_1376 leader sequence.

### Preparation of cells for fluorescence microscopy

For the immunofluorescence microscopy of the adherent cells, wild-type trophozoites were incubated on slides in TYI-S-33 medium for 15 min at 37 °C, fixed in ice-cold methanol for 5 min, and permeabilized in ice-cold acetone for 5 min. The blocking and the immunolabeling steps were all performed in a humid chamber using a solution of 0.25% BSA, 0.25% fish gelatine, and 0.05% Tween 20 in PBS for 1 h each. The cells were stained by an anti-GL50803_9296 antibody produced in rabbit (1:2000 dilution) [97]. The secondary antibody used was Alexa Fluor 488-conjugated anti-rabbit antibody (Life Technologies, 1:1000 dilution) or Alexa Fluor 594-conjugated anti-rabbit antibody (Life Technologies, 1:1000 dilution. After each immunolabeling step, the slides were washed three times for 5 min by PBS supplemented with 0.1% Tween20 (Sigma-Aldrich). Slides were mounted in Vectashield containing DAPI (Vector Laboratories).

In the case of detached cells that were affected by albendazole treatment, the cells were fixed by 1% formaldehyde for 30 min at 37 °C. The cells were then centrifuged at 900×*g* for 5 min at RT and washed in PEM buffer (200 mM PIPES, 2 mM EGTA, 0.2 mM MgSO_4_, pH 6.9). The cells were then resuspended in PEM buffer and transferred to poly-L-lysine-coated coverslips and let to attach for 15 min. The cells were then permeabilized by 0.2% Triton X-100 in PEM buffer for 20 min and washed three times with PEM buffer. Then, the cells were incubated in blocking solution PEMBALG (PEM supplemented with 1% BSA, 0.1% NaN_3_, 100 mM lysine and 0.5% gelatin) for 30 min. All blocking and immunolabeling steps were performed in humid chamber at room temperature. After blocking, the cells were incubated in PEMBALG containing anti-acetylated tubulin antibody produced in mouse (1:2000 dilution, Sigma-Aldrich) and anti-GL50803_9296 antibody produced in rabbit [97] (1:2000 dilution) for 1 h. The coverslips were then washed three times 15 min in 0.1% Tween-20 in PBS and incubated in PEMBALG containing anti-mouse antibody conjugated with Alexa488 (1:1000 dilution, Life Technologies) and anti-rabbit antibody conjugated with Alexa594 (1:000 dilution, Life Technologies) for 1 h. The coverslips were washed three times 15 min in 0.1% Tween-20 in PBS and mounted in Vectashield containing DAPI (Vector Laboratories).

For STED microscopy of gently lysed *Giardia*, the cells were resuspended in SM buffer supplemented with protease inhibitors (Complete, EDTA free, Roche) and passed 10 times through 33 × ½” needle (Cadence Inc.). The cell lysate was then put on Piranha-treated high-performance coverslip (Zeiss). After 15 min, the sample was fixed using 2% formaldehyde for 15 min. The cells were then permeabilized, blocked, and immunolabeled as described above. Secondary antibodies used in this experiment were as follows: anti-rabbit Abberior STAR 635p antibody (1:100 dilution, Abberior Instruments GmbH) and anti-mouse Abberior STAR 580 antibody (1:100 dilution, Abberior Instruments GmbH). Anti-fade liquid Abberior Mount (Abberior Instruments GmbH) was used for mounting the slides according to the manufacturer’s protocol.

For live-cell imaging experiments, *Giardia* trophozoites expressing *Gi*DHFR fused with Y-FAST tag [34] were enriched for mitotic cells as described above. Albendazole-unaffected cells were collected and placed to the 35 mm glass bottom microscopy dish (In Vitro Scientific). Cells were kept at 37 °C for 10 min to attach. After that, warm, fresh media supplemented with 1 μM HMBR (a kind gift from prof. Gautier, Sorbonne University, France) was added to the cells, and they were observed using Leica SP8 confocal microscope in 37 °C-preheated humid chamber (Okolab) under anaerobic conditions.

### Fluorescence microscopy and imaging

Static images were acquired on Leica SP8 FLIM inverted confocal microscope equipped with 405 nm and white light (470–670 nm) lasers and FOV SP8 scanner using HC PL APO CS2 63x/1.4 NA oil-immersion objective. Laser wavelengths and intensities were controlled by a combination of AOTF and AOBS separately for each channel. Emitting fluorescence was captured by internal spectrally tunable HyD detectors. Imaging was controlled by the Leica LAS-X software. Images were deconvolved using SVI Huygens Professional software (Scientific Volume Imaging) with the CMLE algorithm. Maximum intensity projections and brightness/contrast corrections were performed in FIJI ImageJ software.

Live imaging experiments were performed on the same microscope as described above. Y-FAST fluorescence was excited at approximately 480 nm, and emitted light was approximately 540 nm as described in [34]. Fluorescence images were taken every 500 ms.

STED and 3D-DyMIN STED [38] microscopy were performed on a commercial Abberior STED 775 QUAD Scanning microscope (Abberior Instruments GmbH) equipped with Ti-E Nikon body, QUAD beam scanner, Easy3D STED Optics Module, and Nikon CFI Plan Apo 60x oil-immersion objective (NA 1.40). Samples were illuminated by pulsed 561 nm and 640 nm lasers and depleted by a pulsed 775 nm STED laser of 2D donut shape (all lasers: 40 MHz repetition rate). The fluorescence signal was detected with single-photon counting modules (Excelitas Technologies). Line-interleaved acquisition enabled separated detection of individual channels in the spectral range from 605 nm to 625 nm and from 650 nm to 720 nm. The confocal pinhole was set to 1 AU.

### LM and CLEM of *Giardia*

For FIB/SEM experiments, laser marked slides were used (LASERMarking, Munich, Germany) and the cells in a proliferative phase were allowed to attach to them in a silicon anaerobic chamber at 37 °C. Slides were rinsed with PBS, pH 7.2, and immediately fixed with 2.5% (v/v) glutaraldehyde (Science Services GmbH, München) in 75 mM cacodylate (Sigma-Aldrich), 75 mM NaCl, 2 mM MgCl_2_ for 30 min, followed by 3 washing steps in cacodylate buffer. Cells were stained with DAPI, sealed with a coverslip and Fixogum (Marabu GmbH & Co. KG, Tamm, Germany) to prevent drying during LM investigation on Zeiss Axiophot fluorescence light microscope. The positions of cells in the desired stage of mitosis (ROIs) were marked on a template, with the same coordinates. For documentation, epifluorescence, phase contrast, and DIC images were taken in different magnifications (objective × 5, × 10, × 40), and these were sufficient to followingly retrieve the ROIs in SEM.

### EM preparation

After removal of Fixogum and coverslip, cells were post-fixed (customized rOTO-impregnation protocol [39] with 1% (v/v) OsO_4_ and 1% (w/v) K_4_Fe(CN)_6_ in cacodylate buffer for 30 min, washed 3 times in ddH_2_O, incubated with 1% (w/v) thiocarbohydrazide in ddH_2_O for 30 min, washed = with ddH_2_O 3 times, followed by post-fixation with 1% OsO_4_ in ddH_2_O for 30 min. The samples were rinsed 3 times with ddH_2_O and dehydrated in a graded series of acetone (20%, 40%, 60%, 80%, 100%, 100%, 100%), containing a 1% uranyl acetate step in 20% acetone for 30 min, infiltrated and embedded on the glass slide.

The ultrathin embedding was done according to [40] as follows. Cells were infiltrated with 1:1 Hard-Plus Resin-812 in acetone, 2:1 (resin/acetone) and 3:1 (resin/acetone) always for 1 h. Finally, the cells were placed in two changes of 100% resin, for 1 h and 2 h. A filter paper, completely soaked with acetone, was placed at the bottom of a Falcon® tube to provide an acetone saturated atmosphere. A polypropylene cap was placed on top of the filter paper to avoid direct contact with the slide. The slide was placed upright into the Falcon® tube, allowing the excessive resin to drain into filter paper at the bottom of the Falcon tube for 2 min. Then, the slides were centrifuged for 2 min, 268 g, Hettich, swing-out rotor). The samples were polymerized for 72 h at 60 °C. The size of the glass slides was reduced to appropriate size by fracturing with aid of a diamond pen. The specimens were mounted on an aluminum stubs with colloidal silver. A carbon coating by evaporation of approximately 15 nm thickness was done on Balzers BAE 080 T, Liechtenstein).

### High-resolution FIB/SEM

*Giardia* interphase and mitotic trophozoites cells were imaged in a Zeiss Auriga 40 FIB/SEM workstation operating under SmartSEM (Carl Zeiss Microscopy GmbH, Oberkochen, Germany). FIB/SEM milling was started right in front of the anterior part the cell. Ion beam currents of 50 pA were used. High-resolution images were obtained with the EsB detector at 1.5 kV at a grid voltage of − 500 V. Dependent on the desired resolution, image pixel sizes between 2 and 10 nm in x/y were chosen. The milling rate was set to 2 nm, which allows the adjustment of the z resolution in 2 nm steps at any time during the FIB/SEM run. The average voxel size achieved was 3 × 3 × 8 nm.

### 3D-reconstruction and visualization

The datasets were aligned using Amira (Thermo Fisher Scientific, USA) with the module *align slices*. The image stacks from FIB/SEM were segmented and reconstructed in Thermo Scientific Amira software or processed with a direct volume rendering algorithm (volren) for immediate visualization.

## Supplementary Information

**Additional file 1: Movie S1.** Movie of dividing Y-FAST labeled mitosomes.

**Additional file 2: Movie S2.** Movie of 3D STED of central mitosomes.

**Additional file 4: Fig. S1.** Illustrative FIB/SEM pipeline for *Giardia.*

**Additional file 5: Fig. S2.** 3D rendering of FIB/SEM. The exemplary image of central mitososomes in 3D rendering of FIB/SEM images. Use glasses for stereoimaging.

**Additional file 6: Fig. S3.** Connecting microtubular fiber and funis. Longitudinal reconstruction from individual FIB/SEM sections of *Giardia* interphase cell showing funis - the axial cytoplasmic microtubular cytoskeleton (white arrowheads). It accompanies the pair of caudal flagella both from ventral (A) and dorsal (B) sides. Individual microtubules radiate laterally from the central funis, and also a particular microtubular accumulation called median body (asterix) is coupled to funis microtubules. It is likely that the two funis dorsal and ventral fibers come in close contact by the end of telophase to stabilize the newly established caudal flagella pair after the flagellar transformation during mitosis. TEM transverse sections from different positions in the cell (C, D) show chains of 3-4 microtubules between two caudal flagella (red arrowhead). It is likely that these microtubules establish the mitosomal connector in the proximal part of the cell.

**Additional file 7: Fig. S4.** Reconstruction including the peripheral mitosomes. The peripheral mitosomes shown in white.

## Data Availability

All data generated or analyzed during this study are included in this published article and its supplementary information files. The raw microscopy datasets are available from the corresponding authors on request.
