# Supplementary figures and images for: Inheritance of the reduced mitochondria of Giardia intestinalis is coupled to the flagellar maturation cycle
Source: BMC Biol. 2021 Sep 7;19:193. doi: 10.1186/s12915-021-01129-7 (PMC8422661; doi:10.1186/s12915-021-01129-7)

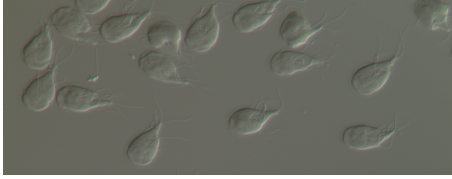

DIC

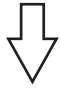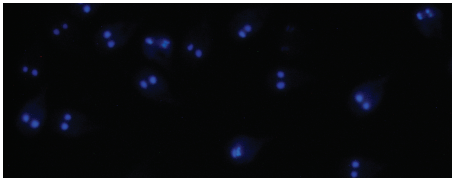

DAPI

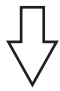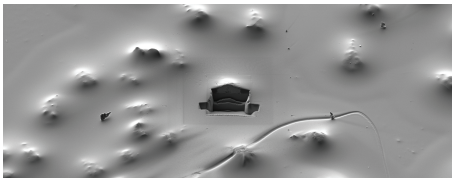

flat embedding - FIB/SEM

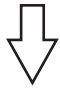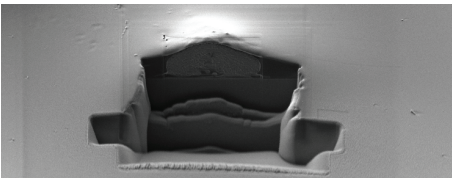

FIB/SEM

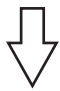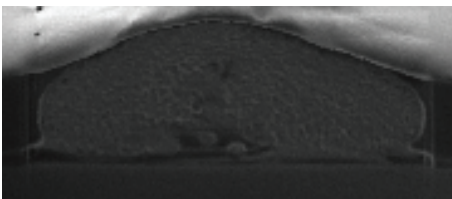

FIB/SEM

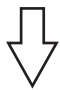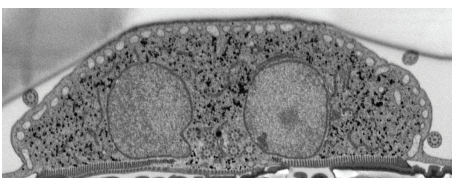

FIB/SEM

Supplement: Supplementary file 5 — Additional file 4: Fig. S1. Illustrative FIB/SEM pipeline for Giardia. [file 12915_2021_1129_MOESM4_ESM.pdf]

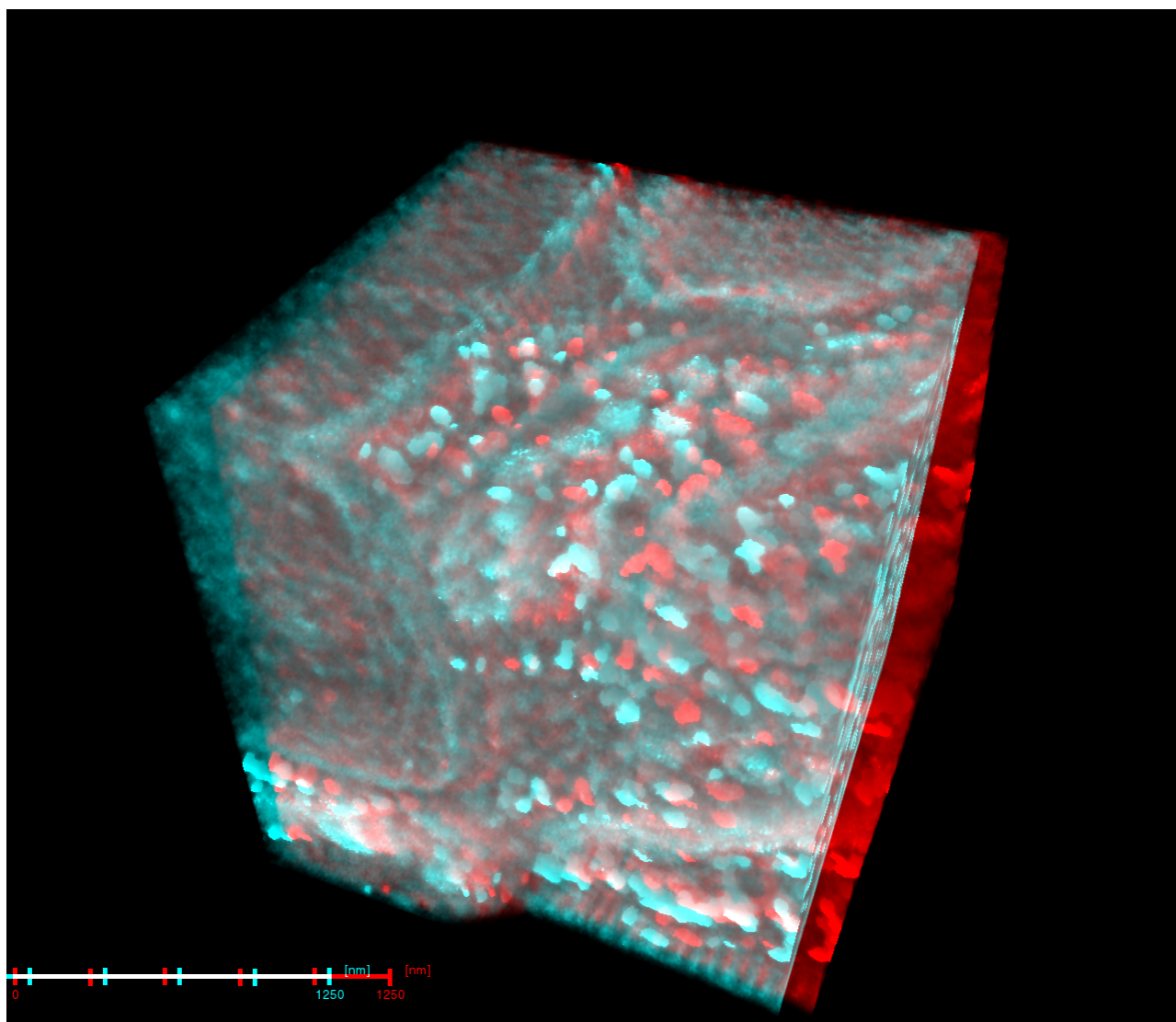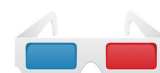

Supplementary File 5

Supplement: Supplementary file 6 — Additional file 5: Fig. S2. 3D rendering of FIB/SEM. The exemplary image of central mitososomes in 3D rendering of FIB/SEM images. Use glasses for stereoimaging. [file 12915_2021_1129_MOESM5_ESM.pdf]

A

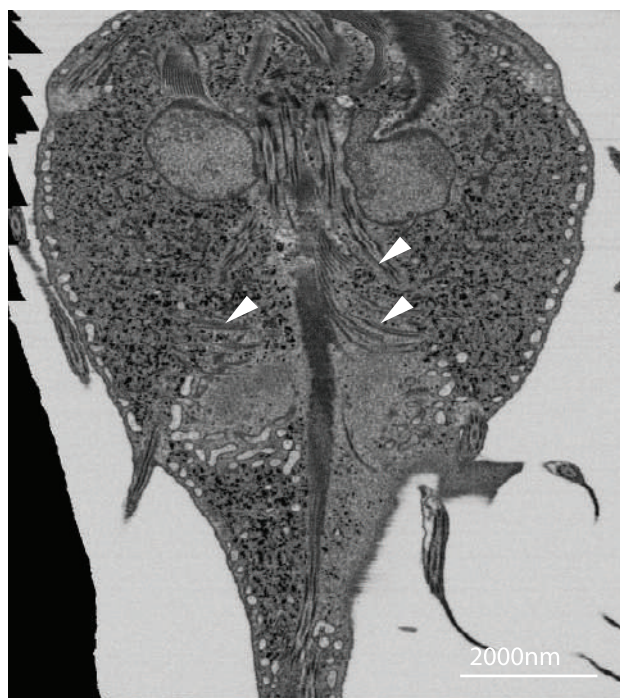

B

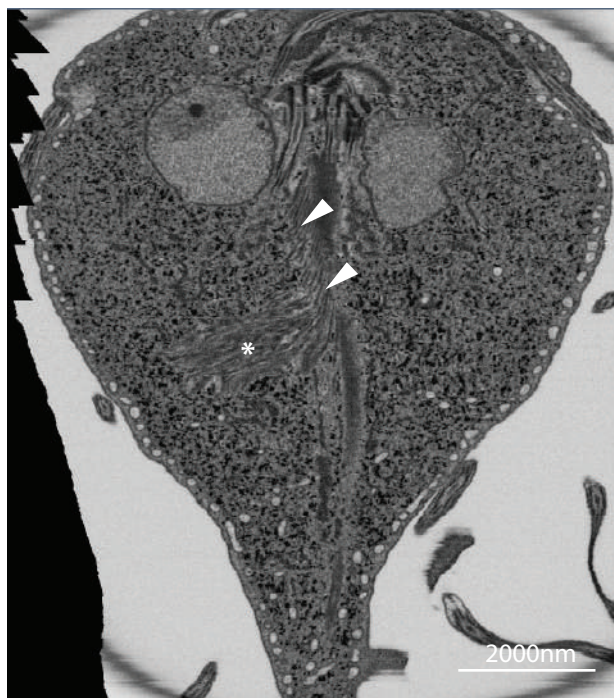

C

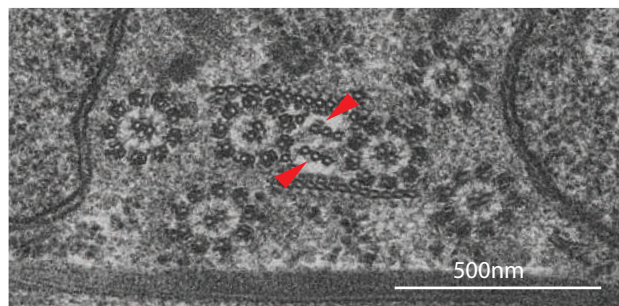

D

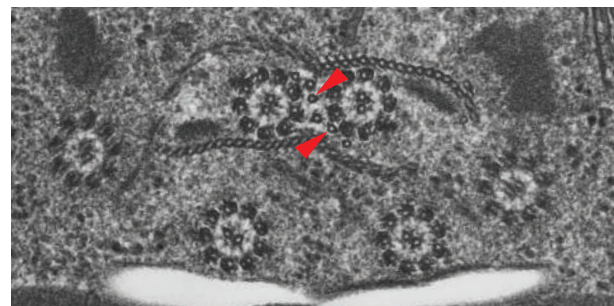

Supplement: Supplementary file 7 — Additional file 6: Fig. S3. Connecting microtubular fiber and funis. Longitudinal reconstruction from individual FIB/SEM sections of Giardia interphase cell showing funis - the axial cytoplasmic microtubular cytoskeleton (white arrowheads). It accompanies the pair of caudal flagella both from ventral (A) and dorsal (B) sides. Individual microtubules radiate laterally from the central funis, and also a particular microtubular accumulation called median body (asterix) is coupled to funis microtubules. It is likely that the two funis dorsal and ventral fibers come in close contact by the end of telophase to stabilize the newly established caudal flagella pair after the flagellar transformation during mitosis. TEM transverse sections from different positions in the cell (C, D) show chains of 3-4 microtubules between two caudal flagella (red arrowhead). It is likely that these microtubules establish the mitosomal connector in the proximal part of the cell. [file 12915_2021_1129_MOESM6_ESM.pdf]

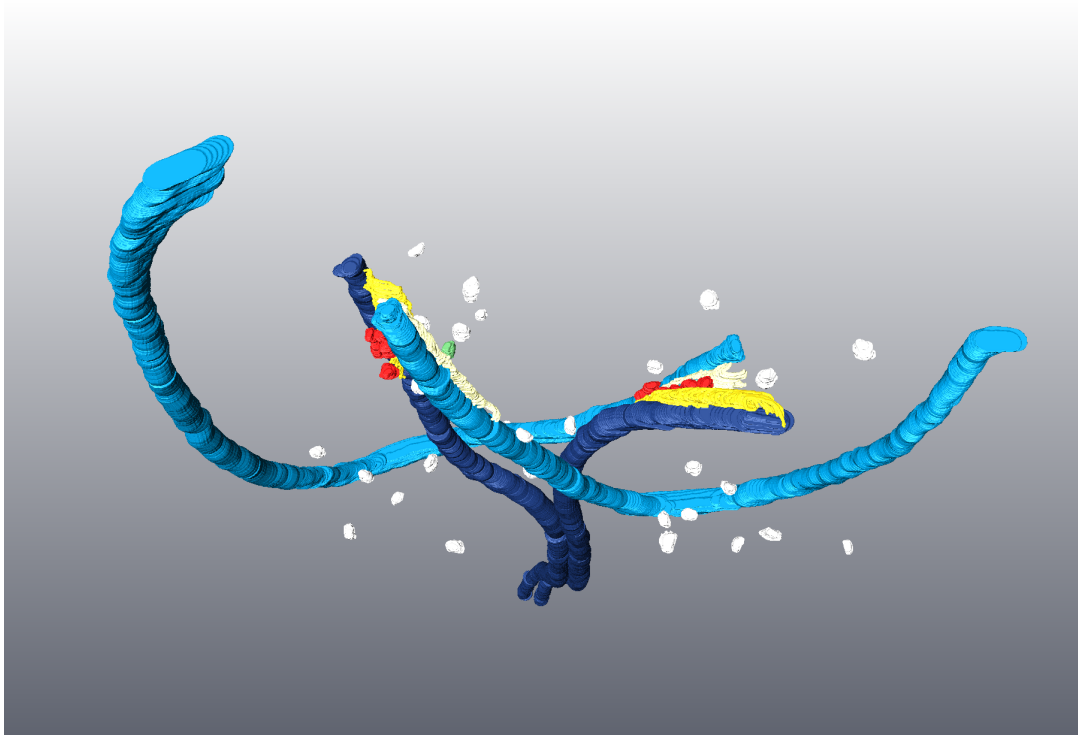

Supplementary File 7

Supplement: Supplementary file 8 — Additional file 7: Fig. S4. Reconstruction including the peripheral mitosomes. The peripheral mitosomes shown in white. [file 12915_2021_1129_MOESM7_ESM.pdf]
